# Study on Genetic Transformation System of Cabbage (*Brassica oleracea* var. *capitata*) Based on Transgenic Root Regeneration

**DOI:** 10.3390/plants14243754

**Published:** 2025-12-10

**Authors:** Meiqi Tao, Wenlong Wang, Xi Shan, Zhongliang Dai, Changwei Zhang, Zhenchao Zhang

**Affiliations:** 1 Zhenjiang Institute of Agricultural Sciences in Hilly Area of Jiangsu Province, 1# Hongjing Road, Jurong 212400, China; 20212804@jaas.ac.cn (M.T.); shanxi@jaas.ac.cn (X.S.); daizhongliang@jaas.ac.cn (Z.D.); 2 State Key Laboratory of Crop Genetics & Germplasm Enhancement and Utilization, Nanjing Agricultural University, Nanjing 210095, China; 2023804298@stu.njau.edu.cn (W.W.); changweizh@njau.edu.cn (C.Z.)

**Keywords:** cabbage (*Brassica oleracea* var. *capitata*), CRISPR/Cas9, transgenic root regeneration, *PUB13*

## Abstract

Cabbage (*Brassica oleracea* var. *capitata*) is a biennial plant. Gene editing technology has not been extensively studied in this species. In this study, we report the induction of highly efficient and heritable transgenic roots in cabbage using the CRISPR/Cas9 (Clustered Regularly Interspaced Short Palindromic Repeats/CRISPR-associated protein 9) gene editing system, followed by the regeneration of whole plants from these transgenic roots. We designed and constructed a CRISPR/Cas9 gene editing vector targeting the immune regulatory gene *PUB13* (*PLANT U-BOX 13*) and introduced it into plants through *Agrobacterium*-mediated transformation. By performing targeted mutations on *PUB13*, transgenic roots were obtained, the optimal TDZ (Thidiazuron) concentration for bud induction (0.9 mg·L^−1^) was determined, and then an efficient transformation protocol from transgenic roots to plants was established, leading to the regeneration of gene-edited plants. In summary, we successfully generated gene-edited cabbage (*Brassica oleracea* var. *capitata*) plants through *PUB13* gene mutagenesis using an innovative transgenic root regeneration approach. A new pathway for obtaining gene-edited cabbage plants was established.

## 1. Introduction

Over the past few decades, crop phenotypic improvements have primarily derived from random mutations induced by chemical or physical mutagenesis [1]. Genetic modification technology serves as a cornerstone research tool for unraveling the mysteries of gene function and cultivating crops with desirable traits. This technology enables the introduction of target genes or suppression of endogenous gene expression (e.g., via RNA interference) through the random insertion of exogenous gene sequences into the plant genome. With the advancement of technology, the emergence of genome editing tools has enabled precise modifications at specific genomic loci. This groundbreaking technology enables precise gene insertion, targeted gene replacement, and directed disruption of specific gene functions, offering unprecedented technical support for crop genetic improvement [2,3]. The emergence of targeted gene editing technologies has significantly improved the process of mutant generation. Rapid and efficient plant genome editing can accelerate both gene function studies and crop improvement. The type II clustered regularly interspaced short palindromic repeats (CRISPR)-associated protein (Cas) system (CRISPR/Cas9) offers high efficiency, low cost, simple design requirements, and cost-effectiveness [4]. Gene disruption efficacy has been validated in multiple plant systems including *Hordeum vulgare* (barley), *Arabidopsis thaliana*, *Solanum lycopersicum* (tomato), and *Brassica oleracea* (cabbage) [1,5,6]. CRISPR-Cas technology has been employed to introduce agronomically important traits across multiple crop species. A series of CRISPR-Cas-derived editors enable more precise genome manipulation, enhancing agronomic traits and revolutionizing breeding technologies [7,8].

Currently, the mainstream methods for plant genetic transformation primarily include *Agrobacterium*-mediated transformation, particle bombardment (gene gun method), and protoplast transformation. *Agrobacterium*-mediated transformation exploits the natural infection mechanism of the soil bacterium *Agrobacterium tumefaciens*, making it the most straightforward and versatile preferred method for achieving genetic transformation [5]. *Agrobacterium rhizogenes* is a Gram-negative soil bacterium characterized by its root-inducing (Ri) plasmid, which stimulates the production of hairy roots in plants. In plant genetic transformation, *Agrobacterium rhizogenes* (*R. rhizogenes*) demonstrates higher transformation efficiency than *Agrobacterium tumefaciens* (*A. tumefaciens*) for most species [9,10,11]. For plant species recalcitrant to *Agrobacterium tumefaciens*-mediated transformation, the *Agrobacterium rhizogenes*-based hairy root system represents an effective alternative strategy for genetic studies and biotechnology applications [12]. *R. rhizogenes*-mediated genetic transformation is widely applicable for transforming diverse plant species (e.g., citrus, radish, peach, etc.) [13,14,15]. *Agrobacterium rhizogenes*-mediated transformation offers the advantage of a short operational cycle, and the transgenic hairy roots it induces exhibit rapid growth. This high efficiency significantly enhances the practicality of *Agrobacterium rhizogenes*-mediated transformation for research in metabolite synthesis and functional genomics in plants [16,17]. Hairy root-inducing *Agrobacterium rhizogenes*-mediated genetic transformation is routinely employed to generate transgenic hairy roots [18]. Although it exhibits high transformation efficiency and stability, the differentiation of hairy roots into shoots remains technically challenging. Overcoming the challenge of hairy root-to-shoot differentiation will accelerate gene functional studies and promote wider application of *Agrobacterium rhizogenes*-mediated genetic transformation in plants. Therefore, the issue of hairy root-to-shoot differentiation urgently requires resolution.

Plants and animals employ innate immune systems to recognize and defend against pathogen infection. Accumulated evidence has revealed that numerous PUB proteins perform essential functions in plant innate immunity [19,20]. PUB proteins play crucial roles in plant innate immunity, programmed cell death, and flowering time regulation. In rice (*Oryza sativa*), PUB15 was identified as a regulatory factor that mitigates reactive oxygen species (ROS) stress and suppresses cell death [21]. The study further revealed that the rice PUB family protein Spotted Leaf 11 (SPL11), functioning as an E3 ubiquitin ligase, mediates monoubiquitination of its interacting partner SPIN1 (SPL11-Interacting Protein 1) to suppress SPIN1 activity, thereby positively regulating flowering time [22]. *PUB13* negatively regulates flowering time in *Arabidopsis thaliana*. Concurrently, *PUB13* negatively regulates FLS2-mediated PTI (PAMP-Triggered Immunity) responses, functioning as an immunosuppressive factor [23,24,25]. Although experimental studies on the PUB genes in cabbage are still insufficient, the high homology with their *Arabidopsis* homologs strongly supports the conservation of their function in theory, providing a solid theoretical basis for conducting in-depth research on this gene family.

Auxins and cytokinins play crucial roles in normal growth, differentiation, and morphological maintenance of cells during plant tissue culture. In addition to natural endogenous hormones, a variety of highly bioactive synthetic plant growth regulators (PGRs) have been developed and widely applied. TDZ was introduced into plant cell culture systems as a highly effective cytokinin-like compound, primarily used to induce adventitious shoot regeneration and somatic embryogenesis [26,27]. TDZ has important applications in multiple research fields. For example, as an inducer, TDZ is used to enhance plant regeneration efficiency and gene expression analysis in blueberries and peonies [28,29]. In the construction of genetic transformation systems, TDZ also plays a key role: studies have shown that it can efficiently induce shoot regeneration in strawberries and demonstrates excellent shoot induction ability during the optimization of the gene editing system in willows [30,31].

The CRISPR/Cas9 system has evolved into a powerful tool for plant trait improvement through targeted genome editing [32,33]. Gene editing applications remain relatively limited in *Brassica oleracea* (e.g., cabbage) and other cruciferous plants, with only a few successful genome-editing cases reported to date [34,35,36]. Notably, research on transgenic hairy root induction and subsequent plant regeneration remains an entirely unexplored area. The genetic transformation system successfully established in this study provides a novel technical platform for this species, which is expected to significantly promote the application of genetic transformation techniques in cabbage and other cruciferous crops, thereby substantially advancing broader research in genetic transformation. Furthermore, while the *PUB13* gene is known to function in both immune regulation and growth control, no CRISPR/Cas9-mediated gene editing of *PUB13* has been reported in cabbage to date. This study pioneers the targeting of *PUB13* for gene editing, with the potential to generate novel transgenic plant materials exhibiting enhanced adaptability and delayed bolting. This approach holds significant promise for developing new cabbage germplasm with improved broad-spectrum disease resistance or late-bolting traits, thereby providing valuable resources for disease-resistant breeding and growth period optimization. In this study, we designed sgRNAs targeting two homologous genes of *Brassica oleracea* *PUB13* and constructed CRISPR/Cas9 gene editing vectors. Transgenic roots were successfully generated through genetic transformation. Furthermore, we investigated the regeneration of whole transgenic plants by inducing hairy root-to-shoot conversion using optimized concentrations of the plant growth regulator TDZ. In summary, we successfully established *Brassica oleracea* (cabbage) transgenic root systems via CRISPR/Cas9-mediated genome editing, and ultimately obtained whole transgenic plants through root-derived shoot regeneration. This study pioneered a novel *Agrobacterium rhizogenes*-based genome editing platform for *Brassica oleracea* (cabbage), creating an integrated “CRISPR–hairy-root–plant regeneration” pipeline. Provides a reference genetic transformation protocol for Brassicaceae crops and other recalcitrant species.

## 2. Results

### 2.1. Regeneration System for Whole Plant Recovery from Transgenic Hairy Roots

The RUBY (Red reporter-Based Visualization system) red reporter system was employed as a visual marker for transgenic hairy roots, enabling rapid screening of positive transformants. This experiment systematically evaluated the induction efficiency and morphogenetic effects of TDZ on shoot regeneration from transgenic roots (Table S2), providing both theoretical and practical support for the optimization and application of this regeneration system.

**Figure 1:**
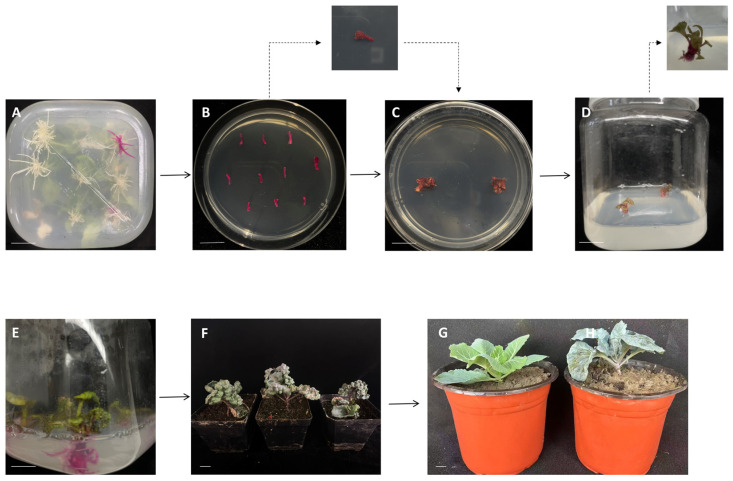
Transgenic root-induced regeneration plant process. (**A**) Transgenic roots obtained via genetic transformation; (**B**,**C**) Bud regeneration induced from transgenic root systems; (**D**) Elongation growth of adventitious shoots; (**E**) Rooting induction for seedling establishment; (**F**) T_0_ transgenic seedlings at approximately 30 days of growth; (**G**) Transgenic plant (**right**) and wild-type plant (**left**) were compared after being transplanted into the field. (**A**–**C**) Approximately 15 days; (**C**–**E**) Approximately 30 days; (**F**) Seedlings at approximately 30 days. (Scale bar = 20 mm).

Following approximately 20 days of culture, red transgenic roots gradually emerged from the wound sites, achieving a transformation efficiency of 25%. After approximately 15 days of culturing transgenic root segments on regeneration medium, compact callus structures began to form at the incision sites of the explants. Subsequently, red bud primordia gradually differentiated on the callus surface and further developed into distinct adventitious shoots (Figure 1B,C). Studies indicated that TDZ concentration significantly influences the process of shoot regeneration from transgenic roots. Through comparative screening, 0.9 mg·L^−1^ was identified as the optimal TDZ concentration for inducing adventitious shoot regeneration from transgenic roots, achieving a maximum shoot regeneration rate of 83.3% under this condition. The entire process from the induction of transgenic hairy roots expressing the RUBY reporter gene, through callus formation, shoot regeneration, and root culture, to the regeneration of complete plants (Figure 1). Ultimately, T_0_ generation transgenic plants carrying the RUBY marker were successfully obtained. Following a nearly three-month process to obtain transgenic plants, no phenotypic differences were observed among the various edited lines. The plants were transplanted to the field for cultivation (Figure 1G) once they reached the growth stage shown in Figure 1F (approximately 30 days old).

### 2.2. Detection of Cas9 Transgene

**Figure 2:**
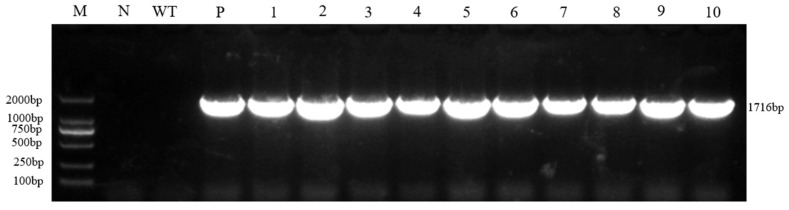
*Cas9* fusion gene PCR detection. M: DL 2000 marker; N: Water; WT: Wild type; P: Positive plasmid; 1~10: Resistant plant.

To verify the successful integration of the vector gene in the regenerated plants selected via the Ruby red marker, molecular identification of the obtained regenerated lines was performed using the *Cas9* gene contained in the vector as the detection target. The results showed that all regenerated plants produced specific bands consistent with the expected size (1716 bp), while no such band was observed in the wild-type control, and the positive plasmid control exhibited the expected amplification signal. These findings indicate that all regenerated plants selected based on the red marker are transgenic positive and successfully carry the target vector sequence (Figure 2).

### 2.3. CRISPR/Cas9-Mediated Gene Editing Results

**Figure 3:**
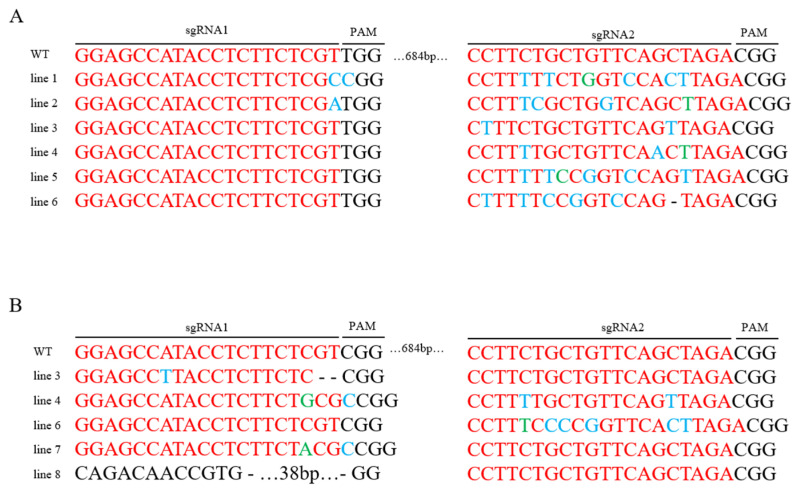
Gene Editing Results. (**A**) Gene *Bol018774*; (**B**) Gene *Bol041392*; Black: PAM (Protospacer Adjacent Motif) sequence; Red: sgRNA target site; Blue: Base substitutions; Green: Base insertions; “-”: Base deletions; Lines 1–8: Gene-edited plant variants; WT: wild-type; sgRNA1 and sgRNA2: two target sites within the *PUB13* gene; PAM sequence: denoted in black on the figure.

Sanger sequencing of PCR products revealed targeted mutagenesis at the *PUB13* locus in a subset of regenerated plants. Consistent with the expectation of obtaining multiple types of gene edits. Validation results demonstrate that the regeneration system enables efficient acquisition of target plants, demonstrating significant utility for both functional gene characterization and precision breeding applications. The gene editing efficiency was determined to be 50%. Various mutation types were detected at the target loci among transgenic-positive plants. The induced mutations comprised three distinct classes: base deletions, insertions, and substitutions. These results demonstrate that the established regeneration system can efficiently generate plants with heritable modifications at the target gene loci, providing a robust technical platform for functional genomics studies and the creation of novel germplasm (Figure 3).

## 3. Discussion

In the relentless pursuit of crop improvement, physical and chemical mutagenesis are being progressively superseded by emerging gene editing technologies, which now represent precise and efficient molecular breeding approaches. Nuclease technologies with programmable targeting capabilities have fundamentally revolutionized genome editing methodologies, thereby driving transformative advances in functional genomics and precision genetic regulation [37,38]. Genome editing relies on site-specific nucleases to introduce one or more breaks in the DNA at the target locus. These breaks are subsequently repaired by the cell’s endogenous DNA repair mechanisms, and imperfect repair can lead to mutations or deletions in the target gene [39]. The clustered regularly interspaced short palindromic repeats (CRISPR) and CRISPR-associated protein 9 (Cas9) system has been extensively utilized for genetic engineering in crop improvement [40,41]. This system plays pivotal roles in enhancing yield, disease resistance, and quality traits of crops. It has been widely adopted for genome editing across diverse plant species [42,43,44]. CRISPR/Cas-mediated genome engineering offers unprecedented opportunities for crop improvement, surpassing conventional induction methods in simplicity, cost-effectiveness, and speed. In most cases, CRISPR-Cas9, gRNA expression boxes, and screening genes are introduced into plants, and transformants are obtained through selection [45]. In this study, we successfully achieved precise editing of the *PUB13* gene target site using the CRISPR-Cas9 system and obtained explants with multiple mutation types. The knockout of this target gene is expected to facilitate the development of novel cabbage germplasm with enhanced environmental adaptability and delayed bolting characteristics. Despite significant advancements in CRISPR/Cas systems, several persistent challenges remain to be addressed. For instance, the editing efficiency of the CRISPR/Cas system exhibits substantial variation across different plant species. This necessitates continuous optimization of existing methods and the development of more robust, versatile, and flexible editing systems [46,47].

The hairy root induction system has become a preferred platform for plant biotechnology due to its rapid growth characteristics and high genetic stability. As an effective methodology for investigating gene expression and function, this system has been widely adopted across diverse plant species to assess genome-targeting editing efficiency. Major breakthroughs have been achieved in overcoming barriers to crop genetic improvement. In cucumber studies, transgenic hairy roots have been efficiently induced via *Agrobacterium rhizogenes*-mediated transformation [48,49,50]. As a high-demand vegetable crop, heading cabbage (*Brassica oleracea* var. *capitata*) remains a genetically recalcitrant species, with genetic modification yet to achieve widespread application. Meanwhile, the transgenic root-mediated plant regeneration pathway requires further in-depth investigation to achieve reliable implementation. The progressive maturation of transgenic root-induced plant regeneration systems will hold significant implications for advancing plant genetic transformation methodologies.

Auxin and cytokinin play critical roles in regulating the growth and development of crops. To unlock the regenerative potential of plant cells, researchers have successfully synthesized a variety of compounds with inductive activity to date [51]. TDZ (Thidiazuron) has garnered significant attention in plant cell and tissue culture due to its remarkable efficacy. It is capable of independently facilitating diverse regenerative responses across a wide range of plant species, demonstrating broad applicability. Due to its exceptional morphoregulatory capacity, TDZ serves as a highly efficient bioregulator in plant tissue culture and has become a pivotal active compound in this field. It influences multiple parameters in both in vitro and in vivo plant culture processes [52,53]. Treatment with TDZ induces regeneration in a wide range of plant species [54,55]. TDZ is regarded as the most effective synthetic cytokinin in various contemporary plant regeneration systems, significantly enhancing the propagation efficiency of recalcitrant species. Based on the remarkable capacity of TDZ to efficiently induce shoot formation, this study investigated its ability to promote adventitious shoot regeneration from transgenic roots of cabbage (*Brassica oleracea* var. *capitata*) by testing a gradient of TDZ concentrations. Through comparative cultivation and systematic analysis, the optimal TDZ concentration (0.9 mg/L) was determined, ultimately achieving successful regeneration of complete plants from transgenic root systems. These results provide valuable data and technical references for the application of TDZ in shoot induction in cabbage (*Brassica oleracea* var. *capitata*) and other cruciferous crops.

Ubiquitination modification is an important post-translational regulatory mechanism of proteins. It is widely involved in regulating various biological processes such as plant growth and development and disease resistance and immune responses. Plant U-box proteins (PUBs) are the earliest identified E3 ubiquitin ligase family in the plant kingdom, and their U-box domains have shown a high degree of structural conservation during evolution [56,57]. E3 ubiquitin ligases play a key regulatory role in plant growth and development by selectively degrading target proteins. Studies have demonstrated that other members of this family also regulate various plant biological processes through multiple pathways [58]. Furthermore, *PUB13* has been identified as a regulatory component of abscisic acid (ABA) signaling through its modulation of the coreceptor ABI1 (ABA-INSENSITIVE1) [59]. Concurrently, this protein modulates the abundance of the chitin receptor LYK5 (Lysin Motif Receptor Kinase5) [60].

We have successfully obtained intact transgenic plants through a novel genetic transformation method, wherein the plants exhibit red coloration due to the expression of the *RUBY* reporter gene. According to the study by Wang et al. [36], the leaves may display morphological abnormalities. Following the editing of the *PUB13* gene, the gene-edited plants may not exhibit phenotypic differences compared to wild-type plants; however, these edits are expected to confer enhanced environmental adaptability to the plants. In existing crop genetic transformation systems, most studies rely on *Agrobacterium tumefaciens*-mediated techniques. This approach has been successfully applied to optimize transformation efficiency in broccoli [61], as well as in gene overexpression, gene silencing, and CRISPR/Cas9 genome editing in tomatoes [62]. Nevertheless, the technical pathway of obtaining transgenic roots via *Agrobacterium* rhizogenes and subsequently regenerating them into intact plants still requires systematic exploration. To address the aforementioned challenges, this study has successfully established an A. rhizogenes-based genetic transformation method: transgenic roots are first obtained and then induced to differentiate into intact plants. This system opens a new avenue for plant genetic transformation research.

For cabbage crops, the establishment of highly efficient genetic transformation systems has long faced significant challenges due to their generally low transformation and plant regeneration efficiency. Previous studies reported a transformation efficiency of only approximately 13%. In contrast, the integrated “CRISPR–hairy-root–plant regeneration” technical system developed in this study successfully increased the transformation efficiency to 25%, significantly outperforming previously reported levels [63,64]. In plants such as tomato and *Arabidopsis*, *PUB13* has been demonstrated to participate in plant defense responses [65,66]. However, the function of this gene in cabbage (*Brassica oleracea*) remains unexplored. Through targeted knockout of the *PUB13* gene in cabbage, we aim to develop cabbage germplasm with enhanced disease resistance. In summary, we have successfully established a novel genetic transformation system. This system provides an innovative approach and a referable new paradigm for addressing the challenges of genetic improvement in cruciferous crops and other recalcitrant plant species.

## 4. Materials and Methods

### 4.1. Vector Construction

**Figure 4:**
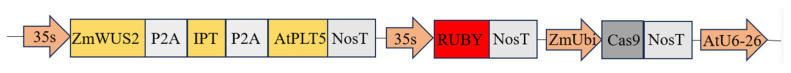
Gene editing vector.

The WIP-RUBY-Cas9 vector, maintained by the Chinese Cabbage Systems Biology Laboratory at Nanjing Agricultural University’s College of Horticulture (Figure 4), was utilized for this study (https://www.molecularcloud.org/plasmid/WIP-RUBY-cas9/MC-0101503.html (accessed on 13 June 2025)) [27]. Based on the two homologous genes of cabbage (*Brassica oleracea* var. *capitata*) *PUB13* (*Bol018774* and *Bol041392*), we designed targeted editing strategies to modify both alleles. Utilizing the CRISPR design tool developed by Huazhong Agricultural University (http://cbi.hzau.edu.cn/crispr/ (accessed on 12 June 2025)), we designed sgRNAs targeting the *PUB13* gene sequence (Table S1). Subsequently, the sgRNA expression cassettes were chemically synthesized in vitro. The expression cassette was inserted into the WIP-RUBY-Cas9 vector via the AscI restriction site to construct the final CRISPR/Cas9 gene editing vector. The vector plasmid was transferred to *Agrobacterium* K599 using the freeze–thaw method. After incubating at 28 °C in a bacterial dark incubator for 2 days, positive clones were picked from the plates and transferred to 1 mL of LB (Lennox Broth) liquid medium containing 50 mg·L^−1^ kanamycin (Kan) and 50 mg·L^−1^ streptomycin (SM) in LB liquid medium and incubated overnight in a dark, 28 °C, 250 r·min^−1^ shaking incubator. PCR validation was performed using carrier-specific primers RUBY-F and RUBY-R (Table S1).

### 4.2. Explant Genetic Transformation

The heading cabbage (*Brassica oleracea* var. *capitata*) seeds used in this study were provided by the Zhenjiang Academy of Agricultural Sciences, Zhenjiang, Jiangsu Province, China.

Explant preparation: A suitable quantity of cabbage (*Brassica oleracea* var. *capitata*) seeds was subjected to a stepwise surface sterilization protocol. The seeds were first immersed in 75% ethanol for 3 min with gentle agitation to ensure full contact, followed by three rinses with sterile distilled water to remove residual ethanol. They were then treated with 15% sodium hypochlorite solution for 10 min under constant shaking, and finally washed five times with sterile distilled water to completely eliminate disinfectant residues. The sterilized seeds were evenly plated on the surface of ½ MS (Murashige and Skoog Medium) solid medium (containing 30 g·L^−1^ sucrose and 7 g·L^−1^ agar, pH adjusted to 5.8) and cultured in a growth chamber under a 16 h light/8 h dark photoperiod at 24 °C for approximately one week.

*Agrobacterium* preparation: Add *Agrobacterium rhizogenes* K599, which has been transformed with the target vector, to liquid LB medium containing antibiotics (50 mg·L^−1^ kanamycin and 50 mg·L^−1^ streptomycin), and incubate overnight at 28 °C on a shaking incubator at 250 rpm·min^−1^. Centrifuge at 6000 rpm for 5 min to precipitate the *Agrobacterium*, discard the supernatant, resuspend the bacterial pellet in MS liquid medium, and adjust the OD600 to 0.6–0.8. Add acetylcinnamaldehyde (150 μmol·L^−1^). Incubate the prepared bacterial suspension in the dark at room temperature for 4 h before use.

Infection and culture: Under sterile conditions in a laminar flow hood, the shoot apex of the seedling was excised approximately 1 cm below the growing point using a sterile scalpel, and the upper stem segment was retained as an explant for *Agrobacterium* infection. The explants were fully immersed in a pre-prepared *Agrobacterium* suspension for 10 min with gentle agitation to ensure uniform infection. After inoculation, the explants were removed, and excess bacterial solution was gently blotted away. They were then transferred to co-culture medium (MS basal salts + 30 g·L^−1^ sucrose + 7 g·L^−1^ agar, pH 5.8) and incubated in darkness at 24 °C for 2 days. Following co-culture, the explants were rinsed thoroughly 3–5 times with sterile distilled water and subsequently transferred to differentiation medium (MS + 30 g·L^−1^ sucrose + 7 g·L^−1^ agar + 200 mg·L^−1^ carbenicillin + 200 mg·L^−1^ timentin) for differentiation culture under a 16 h light/8 h dark photoperiod at 24 °C until shoot regeneration occurred.

Plant regeneration and cultivation: After 15–25 days of differentiation culture, red roots will appear near the incision site of the explant. During tissue culture, well-grown transgenic root segments approximately 1 cm in length were selected as explants. To determine the optimal shoot regeneration conditions, a gradient induction assay was conducted using different concentrations of TDZ. Cut the red roots into small segments and place them on bud induction solid medium with different TDZ concentrations (MS + 30 g·L^−1^ sucrose + 7 g·L^−1^ agar + 200 mg·L^−1^ carbenicillin + 200 mg·L^−1^ timentin + 0.3–1.5 mg·L^−1^ TDZ) for bud induction. Five TDZ concentration gradients were set in an arithmetic sequence, with three replicates per concentration and thirty explants per replicate. Place ten explants in each Petri dish. Using SPSS 26.0 software to conduct error analysis and significance of difference analysis. After bud induction, the red buds are transferred to a shoot culture medium (MS + 30 g·L^−1^ sucrose + 7 g·L^−1^ agar + 200 mg·L^−1^ carbenicillin + 200 mg·L^−1^ timentin) for further cultivation, resulting in positive regenerated plant plantlets. The obtained plant seedlings were then transferred to root induction solid medium (MS + 30 g·L^−1^ sucrose + 7 g·L^−1^ agar + 200 mg·L^−1^ carbenicillin + 200 mg·L^−1^ timentin + 0.1 mg·L^−1^ NAA (1-Naphthaleneacetic Acid) + 0.2 mg·L^−1^ IBA (Indole-3-butyric Acid)) to promote root differentiation. Thirty-five regenerated buds underwent rooting treatment, yielding 30 regenerated plants. After undergoing acclimatization treatment, the plants were transplanted into soil for further cultivation management.

### 4.3. Cas9 Fusion Gene Detection

Transgenic roots showing positive signals based on the Ruby red visual reporting system were selected and regenerated into whole plants. After acclimatization and transplantation, genomic DNA was extracted from young newly emerged leaves of the regenerated plants. Specific primers Cas9-F and Cas9-R (Table S1), designed according to the *Cas9* gene sequence, were used to amplify the *Cas9* gene fragment by PCR. The amplification products were separated by 1.2% agarose gel electrophoresis, and plants showing specific bands of the expected size were identified as transgenic positives. These results confirmed the successful obtainment of transgenic cabbage (*Brassica oleracea* var. *capitata*) plants carrying the *Cas9* gene.

### 4.4. Gene Editing Site Identification

Gene-specific primers *Bol018774*-F/*Bol018774*-R and *Bol041392*-F/*Bol041392*-R (Table S1) were designed based on the sgRNA target regions of two homologous genes, *Bol018774* and *Bol041392*, of the *PUB13* gene in cabbage (*Brassica oleracea* var. *capitata*). Genomic DNA was extracted from TDZ-induced transgenic plants, and the target fragments were amplified by PCR using Taq DNA polymerase. The PCR products were separated by 1.2% agarose gel electrophoresis, and bands of the expected size were purified and sent for Sanger sequencing. The resulting sequencing chromatograms were analyzed using DNAMAN 6.0 software to identify insertions, deletions, or substitutions within the sgRNA target sites. The editing efficiency was calculated using the following formula: Editing efficiency = (Number of gene-edited plants)/(Number of Cas9-positive plants). Transgenic plants carrying intended mutations at the expected sgRNA target sites were selected as positive edited lines for further study.

## Data Availability

The original contributions presented in this study are included in the article/supplementary material. Further inquiries can be directed to the corresponding author.

## Supplementary Materials

The following supporting information can be downloaded at: https://www.mdpi.com/article/10.3390/plants14243754/s1, Table S1: The primers used in this study. Table S2: The effect of different concentrations of TDZ on the regeneration of transgenic roots. Figure S1: Sanger sequencing peak chart results for gene-edited plants.

## Abbreviations

The following abbreviations are used in this manuscript:

TDZ	Thidiazuron
*PUB13*	Plant U-Box 13
NAA	1-Naphthaleneacetic Acid
IBA	Indole-3-butyric Acid
MS	Murashige and Skoog Medium
LB	Lennox Broth
CRISPR	Clustered Regularly Interspaced Short Palindromic Repeats
Cas9	CRISPR-associated protein 9
PAM	Protospacer Adjacent Motif
RUBY	Reporter of Ubiquitous gene expression based on betalain biosynthesis
