# Supplementary material for: Study on Genetic Transformation System of Cabbage (Brassica oleracea var. capitata) Based on Transgenic Root Regeneration
Source: Plants (Basel). 2025 Dec 10;14(24):3754. doi: 10.3390/plants14243754 (PMC12736755; doi:10.3390/plants14243754)
Supplement: Supplementary file 1 [file plants-14-03754-s001.zip › plants-3894622-supplementary.pdf]

Supplementary

Table S1. The primers used in this study.

Oligonucleotides to editing *Bol018774* and *Bol041392*	
sgRNA1	GGAGCCATACCTCTTCTCGT
sgRNA2	TCTAGCTGAACAGCAGAAGG
The primers to detect mutation	
Bol018774-F	GTGGATAGAAGCTGGGCATTCGA
Bol018774-R	ACCACAGAAGCTGCTGATACTTG
Bol041392-F	GTGGTGCGAGGCTAATGACATTG
Bol041392-R	CCACAGAAGCTGCTGACACTTGA
The primers to detect transgenic plants	
Cas9-F	GACAAGAAGTACTCCATCGG
Cas9-R	CTCGATCTTCTTGAAGTAGT
The primers for monoclonal detection	
RUBY-F	TGGATCATGCGACCCTCGCC
RUBY-R	GAACACGGCGATCAGCTTGT

Table S2. The effect of different concentrations of TDZ on the regeneration of transgenic roots.

Code	TDZ concentration (mg · L^{-1})	Number of root segments grafted	Number of roots grow out buds	shoot induction rate
1	0.3	90	45 c	50%
2	0.6	90	63 b	70%
3	0.9	90	75 a	83.3%
4	1.2	90	57 b	63.3%
5	1.5	90	39 c	43.3%

Note: Number of roots grow out buds (Different letters indicate statistically significant differences at $p < 0.05$).

A

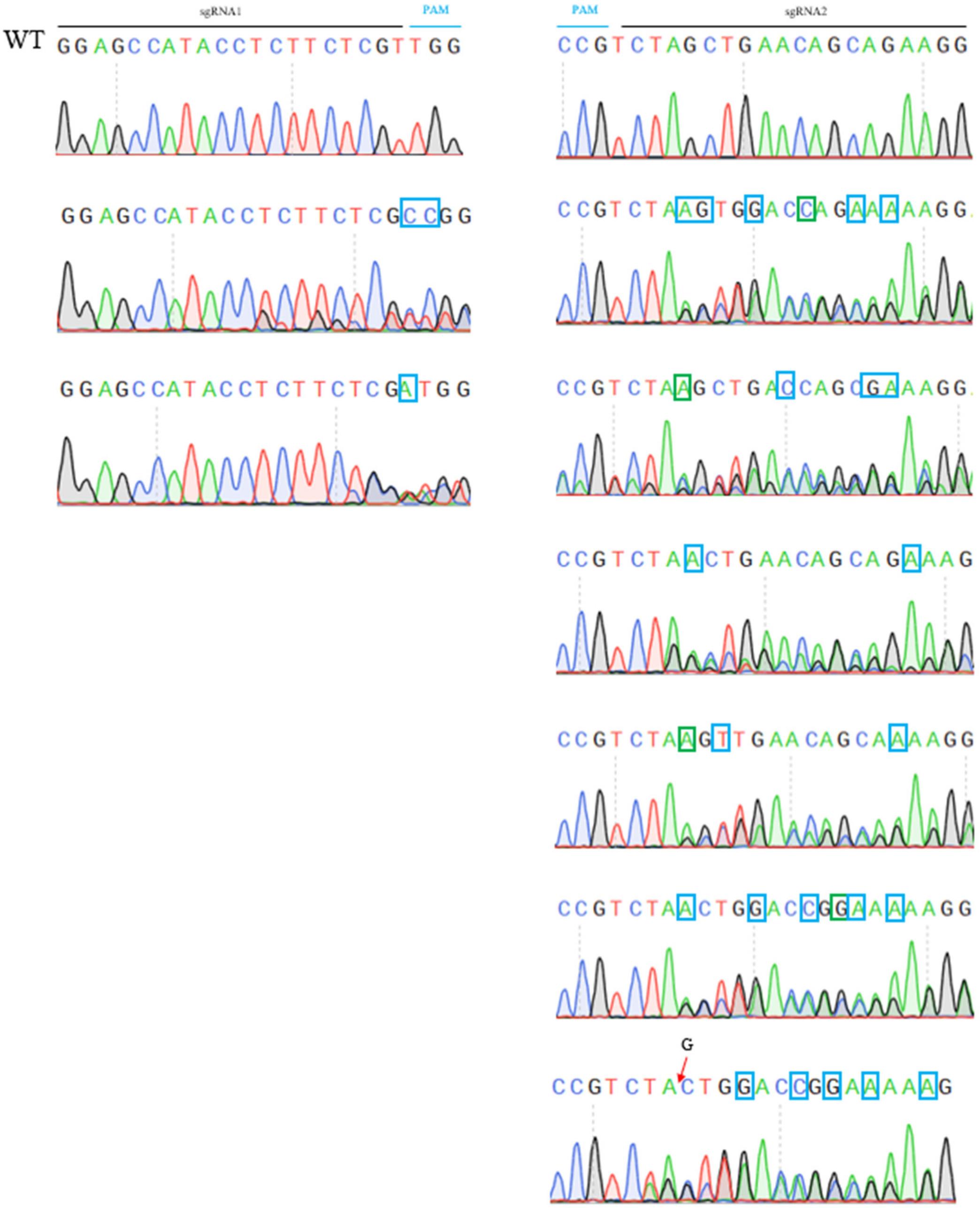
sgRNA1
PAM
PAM
sgRNA2
WT
GGAGCCATACCTCTTCTCGTTGG
CCGTCTAGCTGAACAGCAGAAGG
GGAGCCATACCTCTTCTCGCCGG
CCGTCTAAGTGGACCAGAAAAGG
GGAGCCATACCTCTTCTCGATGG
CCGTCTAAGCTGACCAGCGAAAGG
CCGTCTAACTGAACAGCAGAAAG
CCGTCTAAGTTGAACAGCAAAAGG
CCGTCTAACTGGACCGGAAAAGG
G
CCGTCTACTGGACCGGAAAAAG

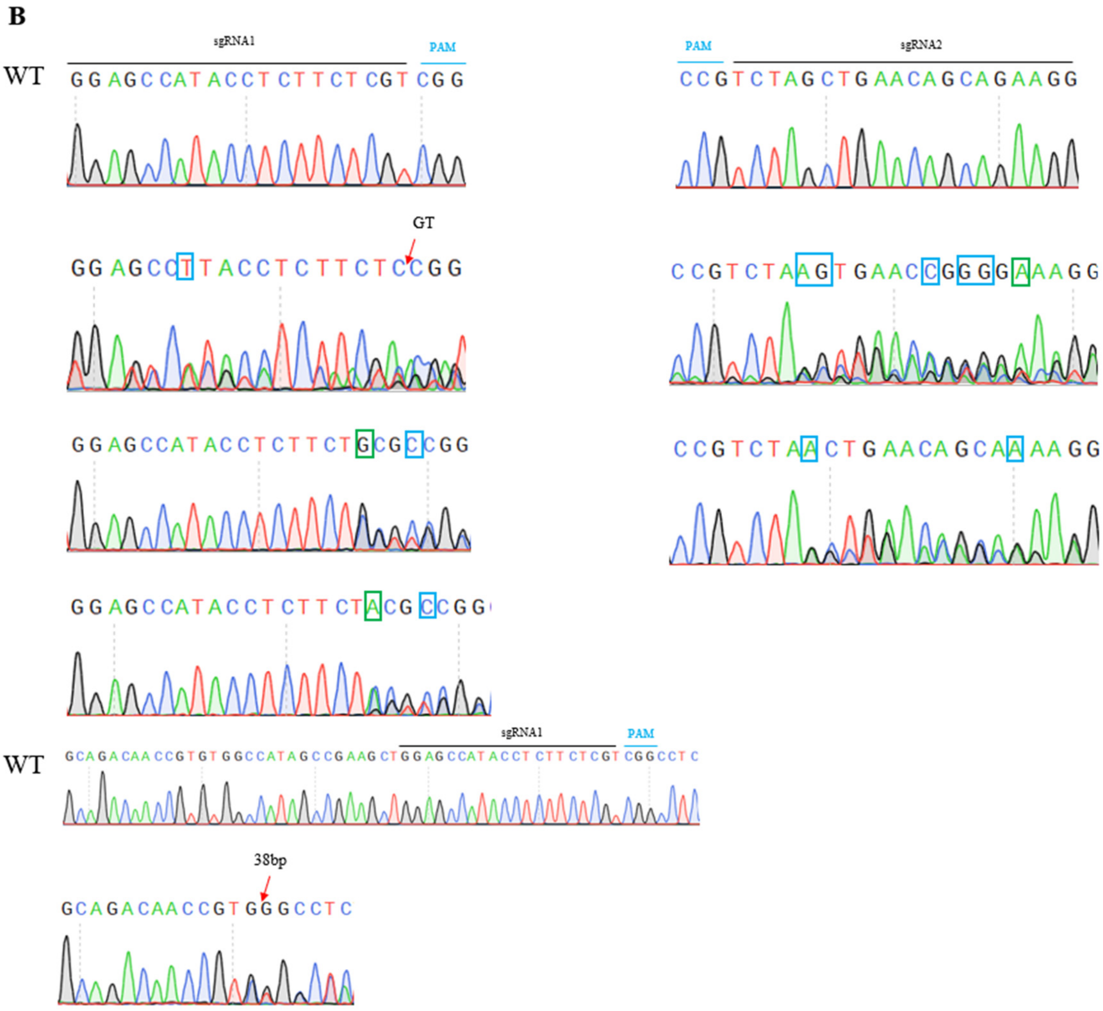

Figure S1. Sanger sequencing peak chart results for gene-edited plants. A: Gene *Bol018774*; B: Gene *Bol041392*; Green box: base insertion; red arrow: base deletion; blue box: base substitution.
